# Protein relative abundance patterns associated with sucrose-induced dysbiosis are conserved across taxonomically diverse oral microcosm biofilm models of dental caries

**DOI:** 10.1186/s40168-015-0136-z

**Published:** 2015-12-19

**Authors:** Joel D. Rudney, Pratik D. Jagtap, Cavan S. Reilly, Ruoqiong Chen, Todd W. Markowski, LeeAnn Higgins, James E. Johnson, Timothy J. Griffin

**Affiliations:** Department of Diagnostic and Biological Sciences, School of Dentistry, University of Minnesota, 515 Delaware St. SE, Minneapolis, MN 55455 USA; Department of Biochemistry, Molecular Biology and Biophysics, University of Minnesota, 321 Church Street SE, Minneapolis, MN 55455 USA; Center for Mass Spectrometry and Proteomics, University of Minnesota, 1479 Gortner Avenue, Saint Paul, MN 55108 USA; Division of Biostatistics, School of Public Health, University of Minnesota, 420 Delaware St. SE, Minneapolis, MN 55455 USA; University of Minnesota Supercomputing Institute, 117 Pleasant St. SE, Minneapolis, MN 55455 USA

**Keywords:** Metaproteomics, Dental caries, Dysbiosis, Sucrose, Microcosm models, Oral biofilm, Taxonomic diversity

## Abstract

**Background:**

The etiology of dental caries is multifactorial, but frequent consumption of free sugars, notably sucrose, appears to be a major factor driving the supragingival microbiota in the direction of dysbiosis. Recent 16S rRNA-based studies indicated that caries-associated communities were less diverse than healthy supragingival plaque but still displayed considerable taxonomic diversity between individuals. Metagenomic studies likewise have found that healthy oral sites from different people were broadly similar with respect to gene function, even though there was an extensive individual variation in their taxonomic profiles. That pattern may also extend to dysbiotic communities. In that case, shifts in community-wide protein relative abundance might provide better biomarkers of dysbiosis that can be achieved through taxonomy alone.

**Results:**

In this study, we used a paired oral microcosm biofilm model of dental caries to investigate differences in community composition and protein relative abundance in the presence and absence of sucrose. This approach provided large quantities of protein, which facilitated deep metaproteomic analysis. Community composition was evaluated using 16S rRNA sequencing and metaproteomic approaches. Although taxonomic diversity was reduced by sucrose pulsing, considerable inter-subject variation in community composition remained. By contrast, functional analysis using the SEED ontology found that sucrose induced changes in protein relative abundance patterns for pathways involving glycolysis, lactate production, aciduricity, and ammonia/glutamate metabolism that were conserved across taxonomically diverse dysbiotic oral microcosm biofilm communities.

**Conclusions:**

Our findings support the concept of using function-based changes in protein relative abundance as indicators of dysbiosis. Our microcosm model cannot replicate all aspects of the oral environment, but the deep level of metaproteomic analysis it allows makes it suitable for discovering which proteins are most consistently abundant during dysbiosis. It then may be possible to define biomarkers that could be used to detect at-risk tooth surfaces before the development of overt carious lesions.

**Electronic supplementary material:**

The online version of this article (doi:10.1186/s40168-015-0136-z) contains supplementary material, which is available to authorized users.

## Background

The lesions of enamel caries can be considered as the outcome of dysbiotic changes in the biofilm community of supragingival dental plaque [1, 2]. Demineralization occurs as the cumulative outcome of repeated shifts towards a less diverse microbiota that produces and tolerates a low pH environment in tooth sites that are sheltered from protective factors in host saliva. Although the etiology of caries is multifactorial, frequent consumption of foods rich in free sugars, notably sucrose, appears to be one of the major factors driving the microbiota in the direction of dysbiosis, particularly in the case of otherwise healthy children with normal salivary flow [3–5].

*Streptococcus mutans* and closely related species (such as *Streptococcus sobrinus*) have long been considered to play a primary etiological role in dental caries. *S. mutans* responds to sucrose by producing large quantities of lactic acid. It is very tolerant of low pH and produces an insoluble extracellular polysaccharide that may sequester acid at tooth surfaces [6]. The mechanisms behind those putative virulence factors have been intensively studied in monoculture [7, 8] and recently in simple multi-species consortia [9]. Much less is known of other species that may also contribute to or protect against dysbiosis driven by dietary carbohydrates [2, 4, 10]. Some strains of oral “non-mutans streptococci” produce and tolerate acid at levels comparable to *S. mutans* [11, 12], while others show increased arginolytic capabilities, which may act to raise pH within the biofilm matrix [13].

*S. mutans* tends to be a minority species even in caries-active children, and carious lesions likewise can occur in children with no detectable *S. mutans* [10, 14–16]. 16S rRNA-based metagenomic comparisons of caries-active and caries-free subjects have detected associations between caries and a variety of oral species, including not only non-mutans streptococci but also members of other genera, such as *Scardovia* and *Bifidobacterium* [10, 14–17]. Caries associations have not been consistent between studies. Moreover, different taxonomic clusters have been defined as subgroups within the same study [16]. This raises an important point. Although caries-associated communities are typically less diverse than healthy supragingival plaque overall, those dysbiotic communities still display considerable taxonomic diversity between affected individuals [10, 14–19]. That in turn raises the question of whether it is desirable to define biomarkers of dysbiosis that are less dependent on taxonomy.

The Human Microbiome project generated comprehensive metagenomic data for a wide variety of body sites in healthy subjects, including supragingival plaque [20]. Although most of that data was based on 16S rRNA sequencing, shotgun metagenomics was also used to catalog the functional potential of all microbial genes within a smaller subset of subjects. One of the key findings was that healthy sites from different people were broadly similar with respect to their functional profiles, even though there was extensive individual variation in their taxonomic profiles [20]. It is possible that the “conservation of function” concept may also extend to dysbiotic communities. This would explain why microbial communities associated with caries still show considerable taxonomic variation. In that case, differential patterns of community-wide gene expression and/or protein relative abundance might provide a more accurate indicator of dysbiosis than can be achieved by counting caries-associated species.

Metatranscriptomic or metaproteomic approaches can be used to provide information on function. A recent metatranscriptomic comparison of subgingival plaque from healthy and periodontally diseased sites in three subjects has provided data that support the “conservation of function” concept. They observed that taxonomically diverse diseased sites shared conserved gene expression profiles [21]. By the same token, a recent metaproteomic comparison of gut microbiotas from healthy controls to Crohn’s disease patients found that major shifts in protein relative abundance by function did not always correlate with changes in taxon relative abundance [22].

In this study, we used an oral microcosm biofilm model of dental caries to investigate differences in community composition and protein relative abundance in the presence and absence of sucrose. This approach provided large quantities of protein, which facilitated deep metaproteomic analysis. Community composition was evaluated using 16S rRNA sequencing and metaproteomic approaches. Although taxonomic diversity was reduced by sucrose pulsing, considerable inter-subject variation in community composition remained. By contrast, functional analysis using the SEED ontology found that sucrose induced changes in protein relative abundance patterns for pathways involving glycolysis, lactate production, aciduricity, and ammonia/glutamate metabolism that were conserved across taxonomically diverse dysbiotic oral microcosm biofilm communities. Collectively, our findings support the concept of using function-based changes in protein abundance as indicators of dysbiosis.

## Results and discussion

### Oral microcosm real-time pH profiles

Direct collection of supragingival plaque requires pooling to obtain adequate amounts of protein for deep shotgun metaproteomics. In our experience, pooling plaque from all sites within a single individual typically yielded only about 1 mg of total plaque by wet weight. Pooling samples from multiple subjects was not a desirable option, since it would have obscured taxonomic diversity between subjects. Accordingly, a previously validated oral microcosm biofilm model was used to scale up protein yields. Microcosms are grown using plaque from a single site as an inoculum, and 16S rRNA studies by members of our group have previously shown that samples taken from the same subjects at different times yield microcosms that are more similar within subjects than between subjects [23]. The oral microcosm approach retains much of the taxonomic variation between individual subjects [23]. Moreover, it simulates many aspects of the caries process, including demineralization at the interface between the tooth and dental restorations [24].

**Fig. 1:**
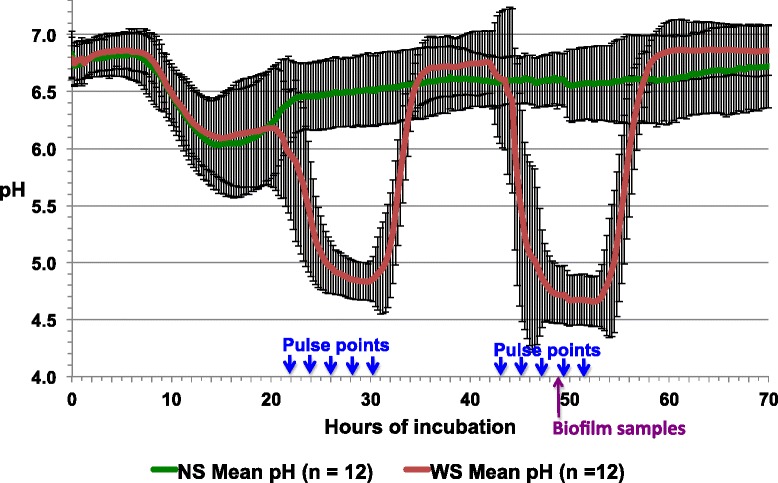
Mean real-time pH curves for paired NS (*green*) and WS (*red*) microcosms from 12 subjects. Readings were taken once every 15 min for 70 h. The *error bars* represent standard deviations. The *blue arrows* denote time points at which the WS microcosms were pulsed with sucrose. The *purple arrow* indicates the approximate time when samples of NS and WS biofilms were collected for protein extraction

Samples of expectorated whole saliva and plaque were collected from each of 12 children at high risk for caries. Each saliva sample was sterilized and used to coat hydroxyapatite disks, which were then inoculated with plaque from the corresponding subject. Inoculated disks were placed into paired CDC biofilm reactors containing basal mucin medium (BMM) [25], and both reactors were incubated at 37 °C under constant shear for 24 h. BMM was then flowed through the “no sucrose” (NS) reactor for 48 h (temperature and shear were held constant). The “with sucrose” (WS) reactor was run in the same way at the same time, but it additionally was sucrose-pulsed five times per day, analogous to three meals and two snacks, for the second and third day. Sucrose pulsing was discontinued at night. Real-time pH was recorded every 15 min, throughout the 72-h incubation. All 12 WS microcosms dropped rapidly below the critical pH for enamel demineralization (pH 5.5) on each day of sucrose pulsing and then rebounded each night (Fig. 1). By contrast, none of their corresponding NS microcosms dropped below critical pH at any time. The WS pH curves showed a cyclical pattern of sucrose-induced acidification similar to those observed in studies of plaque pH in human subjects [26]. This suggested that the microcosm system effectively modeled sucrose-induced dysbiosis.

### Taxonomic diversity within and between NS and WS pairs and original plaque inoculums

No one has yet devised a medium capable of supporting the growth of every oral species. BMM was developed as a saliva analog, with type II hog gastric mucin as the primary carbohydrate source [25]. Additional components intended to promote the growth of oral species include hemin, menadione, urea, and arginine. 16S rRNA sequencing was used to evaluate the extent to which each NS and WS microcosm corresponded to its “parent” plaque inoculum. DNA extracts were sent to the Forsyth Dental Institute Core for Human Oral Microbiome Identification using Next Generation Sequencing (HOMINGS) analysis [27]. The HOMINGS approach parses Illumina Mi-Seq data with a set of validated 16S rRNA sequences (defined as “probes”) to enhance and quantify species- and genus-specific identifications from oral samples.

The complete set of HOMINGS probe counts and relative abundances received from the Forsyth Institute are provided in Additional file 1. Out of 764 distinct HOMINGS probe sequences, 453 were positive in at least one of nine plaque, 12 NS, and 12 WS samples (DNA extracts of plaque samples from subjects 733, 795, and 867 could not be amplified). The average numbers of taxa per sample decreased between the parent plaque inoculums and their corresponding NS and WS microcosms (Table 1). Given that most oral taxa have never been grown in pure culture, and their growth requirements are unknown, it was expected that some might not grow well, even in a complex medium like BMM. On the other hand, it is likely that many oral species may require the presence of partner species in order to grow [15]. In that respect, it is important to note that taxon counts ranged from 79 to 132 for NS microcosms, and 72 to 95 for WS microcosms, and many of the taxa that were retained from the parent inoculums were ones that have not yet been cultured. Thus, although the microcosms were not as diverse as their parent inoculums, they still were much more diverse than any consortium we could have constructed from type strains of cultured species.

**Table 1:** HOMINGS taxon counts for plaque inoculums, NS microcosms, and WS microcosms

	Number of taxa detected^a^			Number of abundant taxa^b^		
Subject	P ^c^	NS^c^	WS^c^	*p* ≥ 1 %	NS ≥ 1 %	WS ≥ 1 %
730	180	79	78	16	12	4
733	ND^d^	84	87	ND^d^	4	4
734	177	132	92	16	10	9
737	199	101	86	19	7	11
760	198	98	72	18	9	12
769	157	103	76	15	8	5
781	178	108	94	22	10	3
795	ND^d^	92	81	ND^d^	7	8
852	165	88	89	16	9	10
861	171	83	88	12	7	10
866	255	102	73	20	7	5
877	ND^d^	92	95	ND^d^	9	8
Mean ± SD	186.6 ± 29.1	96.8 ± 14.3	84.25 + 8.0	17.1 ± 3.0	8.25 ± 2.1	7.4 ± 3.1

^a^Number of taxon probes with a 16S rRNA read count >0

^b^Number of taxa detected at ≥1 % relative abundance (as a percent of total reads per sample) in five or more plaque samples

^c^ *P* plaque inoculums, *NS* no sucrose microcosms, *WS* with sucrose microcosms; these abbreviations remain consistent throughout Tables 1–5

^d^ *ND* not determined; DNA extracts of plaque samples from subjects 733, 795, and 867 could not be amplified

One limitation of the HOMINGS approach is that it will not make taxonomic assignments for reads that do not match any of the defined probe sequences. However, the average percentage of assigned reads across all 33 plaque, NS, and WS samples was 85 %, with a standard deviation of 7.6 %. That was consistent with the oral origins of those samples and suggested that HOMINGS relative abundance estimates for individual taxa were not unduly biased by the presence of unassigned reads (Additional file 1).

**Fig. 2:**
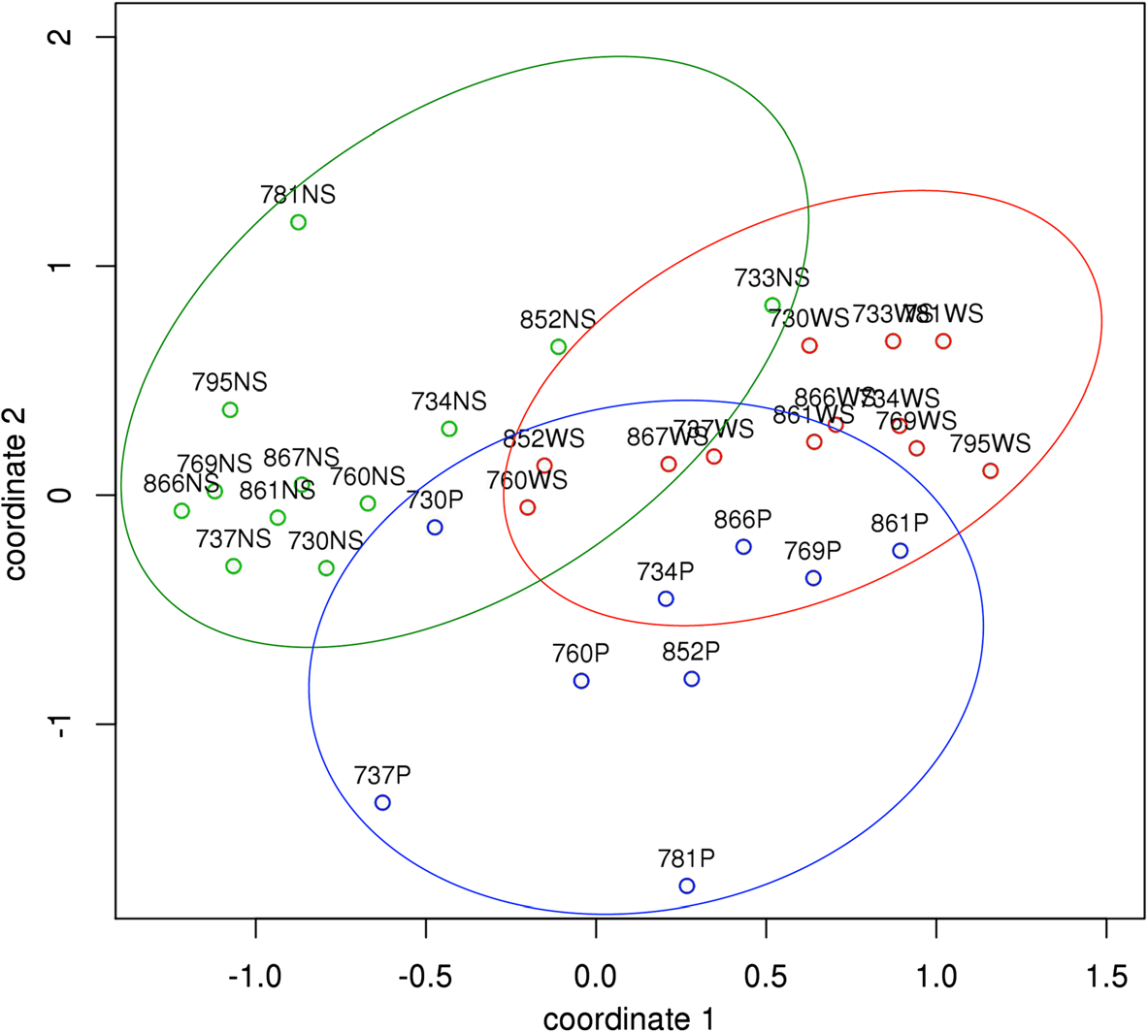
Principal coordinates plot of results for taxonomic data obtained by HOMINGS analysis. The Bray-Curtis distance metric was used. The point labels include the subject number followed by the type of sample. Plaque inoculums (*P*) are shown in *blue*, NS microcosms (*NS*) are shown in *green*, and WS microcosms (*WS*) are shown in *red*. The *blue*, *green*, and *red ellipses* are arbitrary and provided solely to aid visualization of each group

Principal coordinates analysis (PCoA) was used to visualize the Bray-Curtis distances between HOMINGS probe counts for plaque inoculums (P) and their paired NS and WS microcosms (Fig. 2). There was considerable overlap between groups along the coordinate 1 axis, which accounted for the largest proportion of total variance. The plaque inoculums were more distant from the microcosms along coordinate 2, and that might be due to the observed differences in taxon counts between those groups. It is important to note that the plot showed relatively large distances between individual subjects within each of the plaque inoculum, NS, and WS groups, with no tight clusters. This suggested that individual taxonomic variation between plaque inoculums persisted in the microcosms.

The heat map in Additional file 2 graphically displays HOMINGS probe counts for all HOMINGS taxa that were detected in any P, NS, or WS sample. Considerable individual variation was seen within each group. Bray-Curtis hierarchical clustering results for the samples were largely consistent with their spatial distribution within the PCoA plot. Overlaps between sample groups were seen, since 760 WS and 852 WS clustered with the NS samples, while 733 NS clustered with the WS samples. The taxon dendrogram graphically illustrated the pattern of decreasing taxonomic diversity between the P inoculums, NS microcosms, and WS microcosms but also suggested that many of the relatively abundant taxa remained consistent across all three groups. However, other relatively abundant taxa appeared to increase or decrease between the NS and WS microcosms. Those patterns likewise seemed consistent with the patterns of dispersion along coordinates 1 and 2.

Since differences in relative abundance appeared to be exerting a major influence on the PCoA and hierarchical clustering results, we used the edgeR package of the open-source R statistical software package to compare HOMINGS 16S rRNA probe counts between the NS and WS pairs and between the microcosms and their parent inoculums. The edgeR package was developed to analyze count data using generalized linear models (GLM) in conjunction with empirical Bayes estimates of gene-specific dispersions [28]. edgeR incorporates a normalization step and corrects for multiple comparisons by estimating Benjamini-Hochberg false discovery rates (FDR). The GLM approach allows for paired samples, which made it particularly suitable for our study design.

edgeR identified 29 HOMINGS probes (7 % of 426 positive probes) that differed between plaque and NS pairs at FDR ≤ 5 % (a complete list of positive probes and their corresponding FDR values is provided in Additional file 3). Of those with FDR ≤ 5 %, 2 increased in NS, whereas 27 were higher in plaque. However, only 4 of the latter were detected at ≥1 % relative abundance (as a percent of total reads) in five or more plaque samples, Lautropia_mirabilis, Rothia_Genus_probe, Corynebacterium_durum, and Abiotrophia_defectiva. Those probes remained detectable in NS microcosms but at relative abundances <1 %. By contrast, 11 probes meeting the ≥1 % relative abundance criterion above for plaque did not differ from NS at FDR ≤ 5 %. Neither probe that increased in the NS microcosms increased to ≥1 % relative abundance, but both were for *Fusobacterium nucleatum* (Table 2).

**Table 2:** edgeR results for HOMINGS probes abundant in ≥5 samples: NS microcosms vs. plaque

HOMINGS taxon probe^a^	logFC^b^	adjPValue^c^	Abundance >1%^d^
Streptococcus_Genus_probe	1.64	1	9
Neisseria_Genus_probe	7.80	1	8
Rothia_Genus_probe	4.87	1.64E-04*	6
Fusobacterium_Genus_probe	−5.44	1	6
Granulicatella_adiacens_&_paradiacens	−2.07	1	6
Haemophilus_parainfluenzae	−2.46	1	6
Streptococcus_gordonii_&_sanguinis	−1.35	1	6
Veillonella_dispar	−0.96	1	6
Veillonella_Genus_probe	−0.35	1	6
Rothia_aeria	7.97	1	6
Lautropia_mirabilis	9.82	5.25E-05*	5
Abiotrophia_defectiva	2.28	2.87E-03*	5
Corynebacterium_durum	7.97	7.83E-22*	5
Rothia_dentocariosa	4.18	1	5
Gemella_haemolysans	0.63	1	5

^a^Formatted as per HOMINGS output

^b^Log fold-change NS:P

^c^ *p* values adjusted using the Benjamini-Hochberg procedure, controlling the FDR at 5 %; an adjusted *p* value of 1 reflects rounding by Excel of values >0.999; *asterisk* indicates FDR ≤ 5 %

^d^Number of plaque samples in which reads for a probe were ≥1 % (as a percent of total reads)

edgeR identified 80 HOMINGS probes (19 % of 427 positive probes) that differed between plaque and WS pairs at FDR ≤ 5 % (Additional file 4). Of those, 4 increased in WS, whereas 76 were higher in plaque. Only 6 of the probes that were higher in plaque were detected at ≥1 % relative abundance in five or more plaque samples (Table 3). Those were Rothia_Genus_probe, Corynebacterium_durum, Lautropia_mirabilis, Abiotrophia_defectiva, and Gemella_haemolysans. Ten probes meeting the 1 % relative abundance criterion above for plaque did not differ from WS at FDR ≤ 5 % (Table 3). None of the probes that increased in the WS microcosms increased to ≥1 % relative abundance. There were no probes that differed between the NS and WS microcosms with an FDR ≤ 5 % (Additional file 5).

**Table 3:** edgeR results for HOMINGS probes abundant in ≥5 samples: WS microcosms vs. Plaque

HOMINGS taxon probe^a^	logFC^b^	adjPValue^c^	Abundance >1%^d^
Streptococcus_Genus_probe	−2.99	1	9
Neisseria_Genus_probe	8.76	0.38	8
Rothia_Genus_probe	4.20	6.52E-03*	6
Granulicatella_adiacens_&_paradiacens	−2.89	1	6
Haemophilus_parainfluenzae	2.15	1	6
Rothia_aeria	6.30	1	6
Streptococcus_gordonii_&_sanguinis	−3.10	1	6
Veillonella_dispar	−2.68	1	6
Fusobacterium_Genus_probe	2.10	1	6
Veillonella_Genus_probe	−2.67	1	6
Corynebacterium_durum	8.51	<0.00E-163*	5
Lautropia_mirabilis	9.33	1.56E-09*	5
Abiotrophia_defectiva	4.93	5.36E-04*	5
Gemella_haemolysans	4.16	3.15E-03*	5
Rothia_dentocariosa	3.64	1	5

^a^Formatted as per HOMINGS output

^b^Log fold-change WS:P

^c^ *p* values adjusted using the Benjamini-Hochberg procedure, controlling the FDR at 5 %; an adjusted *p* value of 1 reflects rounding by Excel of values >0.999; *asterisk* indicates FDR ≤ 5 %

^d^Number of plaque samples in which reads for a probe were ≥1 % (as a percent of total reads)

*S. mutans* was detected by HOMINGS in seven of the nine plaque samples and 7 of the 12 WS microcosms. Its relative abundance varied considerably but only exceeded 1 % in two plaque samples. *S. mutans* did not exceed 1 % relative abundance in any of the microcosms. It was undetectable in 9 of the 12 NS microcosms (*S. mutans* relative abundance was not significantly different between plaque and WS pairs). *S. sobrinus* was detectable by HOMINGS in four of nine plaque samples, 4 of 12 NS microcosms, and 5 of 12 WS microcosms but only at very low levels. *Scardovia wiggsiae* has recently been proposed as a novel caries-associated species in children [16]. It was detected in five of nine plaque samples but only exceeded a relative abundance of 1 % in one plaque sample. It was present in 5 of 12 NS microcosms and 4 of 12 WS microcosms at relative abundances <1 %. Another proposed caries-associated species, *Bifidobacterium dentium* [16], was detected in only one plaque sample, at a relative abundance well below 1 % (Additional file 1).

Collectively, the HOMINGS results indicated that NS and WS microcosm biofilms retained much of the taxonomic diversity present in their parent plaque samples. Changes did occur, but mostly in taxa that were present at a relative abundance <1 % to start with. It appeared that BMM alone was less supportive of *Rothia*, *Lautropia*, *Corynebacterium*, and *Abiotrophia*, while providing more favorable conditions for the growth of *Fusobacterium* species. However, there is no reason to think that NS microcosms constituted a dysbiotic community with respect to caries, since their pH remained above 6.0. Indeed, some authors have proposed the clinical use of arginine or urea rinses as way of encouraging base production in plaque [13, 29], and both are components of BMM [25]. Moreover, sucrose pulsing clearly simulated caries-like pH drops in WS microcosm biofilms, even though there were no HOMINGS taxonomic differences between NS and WS at FDR ≤ 5 %. This supported our hypothesis that taxonomy alone is not sufficient as an indicator of dysbiosis.

### Taxonomic diversity of peptides within and between NS and WS pairs

A detailed description of our metaproteomic workflow is provided in the “Methods” section. Here, we note only steps that provide context for interpreting the results. Briefly, mass spectra obtained by a separate 2D MSMS analysis of each microcosm were searched against the Human Oral Microbiome Database (HOMD) genomic dataset, using our published two-step strategy [30–33]. Peptide-spectral matches at a 5 % target-decoy search local FDR threshold were used for further analysis (in proteomics, the term “local FDR” refers to the likelihood that an individual protein, peptide, or spectrum has been matched incorrectly in a target-decoy search) [31, 34]. All instances of the spectrum for a given peptide were retained, to allow for spectral counting. Those spectra were searched against the BLAST-NR database, using BLAST-P. The BLAST-P output for each microcosm was parsed using MEGAN5 software, to generate taxonomic assignments and functional analyses [35].

MEGAN5 uses a “Lowest-Common-Ancestor assignment algorithm” (LCA) to assign reads to taxa. Species-specific peptides are assigned at the species level. Conserved peptides with hits assigned to multiple taxa are moved up to higher taxonomic levels in the phylogenetic tree generated by MEGAN5 (e.g., genus, family, phylum, kingdom). The MEGAN5 LCA algorithm assigned 88 % of 1,126,203 total spectra into 592 different taxonomic levels across all 12 NS and WS pairs. The taxon list included 303 species, representing 11 % of normalized spectral counts. Most spectra were moved up to higher taxonomic levels (e.g., genus, family, phylum, kingdom), depending on the extent to which their parent peptides were conserved. That outcome was expected, since sequences that are critical to protein function are less likely to vary across species.

**Fig. 3:**
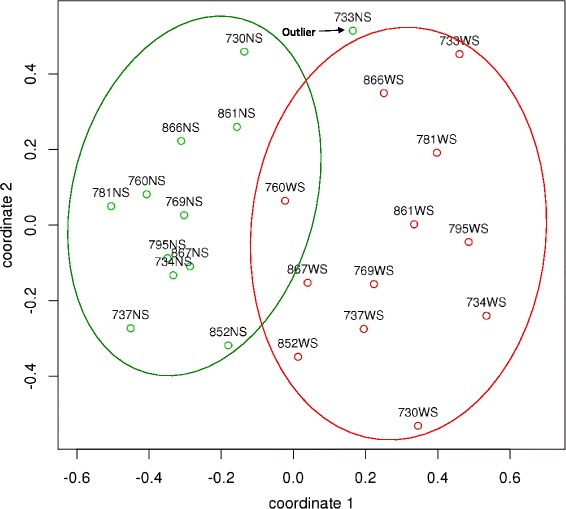
Principal coordinates plot of results for species-level taxonomic assignments by MEGAN5 LCA analysis. The Bray-Curtis distance metric was used. The point labels include the subject number followed by the type of sample. NS microcosms (*NS*) are shown in *green*, and WS microcosms (*WS*) are shown in *red*. The *green* and *red ellipses* are arbitrary and provided solely to aid visualization of each group

Additional file 6 provides the complete list of spectral count assignments at the species level. Figure 3 shows a PCoA plot based on those data. The NS microcosms were separated from the WS microcosms along coordinate 1 (with the notable exception of 733 NS). However, there was wide separation within both the NS and WS microcosms along coordinate 2. This suggested that NS and WS microcosms, although distinguishable on a broad level, did not cluster tightly on the basis of taxonomy. Hierarchical clustering results were quite consistent with the spatial distribution of samples in the PCoA plot, and the heat map likewise showed considerable individual variation in taxonomy within each group (Additional file 7).

edgeR analysis indicated that 24 of the 304 species (8 %) were significantly different between the NS and WS microcosms at FDR ≤ 5 % (Additional file 8). Eleven of those species were abundant at ≥1 % of total spectral counts in 5 or more microcosms. Of those, *Fusobacterium* species *nucleatum*, *periodonticum*, and sp. oral taxon 370 decreased in WS microcosms, as did *Haemophilus parainfluenzae*, *Eikenella corrodens*, and *Abiotrophia defectiva. Streptococcus* species *salivarius*, *vestibularis*, *thermophilus*, and sp. F0442 increased in WS microcosms, as did *Veillonella* sp. ACP1 (Table 4).

**Table 4:** edgeR results for species-level spectra abundant in ≥5 samples: WS vs. NS microcosms

Species assigned to spectra by MEGAN5 LCA	log FC^a^	adjPValue^a^	Abundance >1%^b^
*Haemophilus parainfluenzae*	−7.28	0.02*	13
*Fusobacterium nucleatum*	−7.20	3.90E-09*	10
*Fusobacterium periodonticum*	−10.06	5.76E-06*	10
*Streptococcus salivarius*	6.61	1.65E-06*	9
*Streptococcus sp. F0442*	5.89	0.02*	8
*Fusobacterium sp. oral taxon 370*	−7.96	1.32E-04*	7
*Streptococcus vestibularis*	7.09	6.54E-04*	7
*Streptococcus thermophilus*	5.25	0.04*	7
*Eikenella corrodens*	−8.36	6.22E-04*	5
*Veillonella sp. ACP1*	5.82	0.02*	5
*Abiotrophia defectiva*	−4.54	0.03*	5

^a^Interpretation as per Tables 2–3

^b^This table only lists species combining FDRs ≤ 5 % (*) with relative abundances ≥1 % of the total species-level spectral count in five or more microcosms (13 species with FDRs > 5 % also met the ≥1 % relative abundance criterion—see Additional file 8, rows 26–38)

Thirteen species that met the relative abundance criteria above did not differ between the NS and WS microcosms at FDR ≤ 5 % (Additional file 8). We noted that one of those species, *Bacillus cereus*, was not represented in the current list of HOMINGS probes. However, *Bacillus* species have been detected in the human gut [36], oral mucosa, periodontal pockets, and endodontic infections [37, 38]. In order to verify the presence of *B. cereus* 16S RNA in the original plaque inoculums, the Illumina Mi-Seq FASTQ files generated in the first step of the HOMINGS workflow were parsed with the QIIME metagenomic analysis software package [39]. Six of the nine available plaque inoculums contained reads assigned to *B. cereus* at the species level, at relative abundances ranging from 0.003 to 0.79 % (mean = 0.17 %). NS and WS microcosms grown from those inoculums likewise were positive for *B. cereus* 16S RNA.

*S. mutans* spectral counts were detected in only 4 WS microcosms (at low levels), whereas HOMINGS detected it in 7 WS microcosms. *S. sobrinus* spectra were detected in 11 WS microcosms, while HOMINGS detected it (at low relative abundance) in only 5 WS microcosms (Additional files 1 and 8).

Collectively, the LCA results indicated that taxon-specific protein relative abundance patterns only partially corresponded with relative abundance estimates based on 16S rRNA counts. That was not surprising, since HOMINGS used only data from the V3–V4 hypervariable regions of 16S rRNA, while the LCA used data derived from a search of all genes in the BLAST-NR database. The absence of tight NS and WS clusters in the LCA PCoA plot and heat map further suggested that taxonomic information may not be the most effective way to identify microbial communities that have become dysbiotic due to frequent exposure to sucrose.

### Conservation of function within and between NS and WS pairs

MEGAN5 performs functional analyses by mapping associated RefSeq IDs in BLAST-P output files to functional roles within the SEED, COGs, or KEGG ontologies. COGs is no longer being maintained by NCBI, and most KEGG pathway maps are based on the human genome. SEED is specific for prokaryotes [40], so it seemed the best choice for functional analysis of each metaproteome.

The SEED ontology organizes proteins into metabolic subsystems, which in turn are grouped into major functional categories (carbohydrate metabolism, amino acid metabolism, etc.). Our original intent was to compare SEED assignments for NS and WS microcosms at the subsystem level. However, many proteins were binned into more than one subsystem, an inevitable consequence of overlap between metabolic pathways. This raised the question of whether differences between NS and WS microcosms at the subsystem level might be attributable to a common set of proteins. We also observed that changes in the relative abundance of component proteins within subsystems sometimes went in opposite directions (some were higher in NS, whereas others were elevated in WS).

We decided to analyze the SEED output at the level of individual proteins, in order to facilitate interpretation of the results. Since most spectra were assigned to higher taxonomic levels by MEGAN5 (see above), the findings reported below should be interpreted as a composite picture of responses by taxonomically diverse microcosms to sucrose pulsing.

**Fig. 4:**
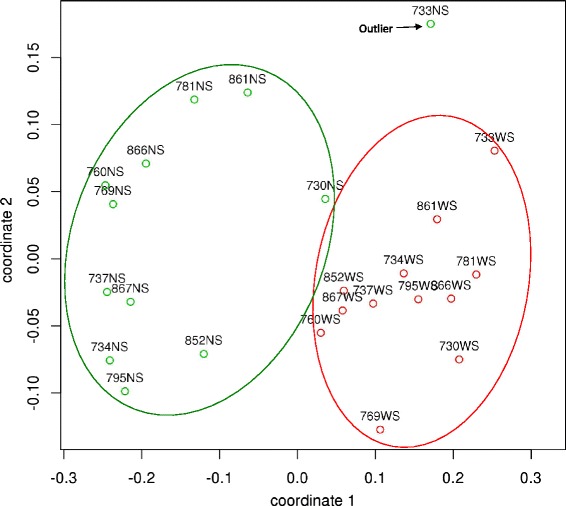
Principal coordinates plot of results for proteins identified by MEGAN5 SEED analysis. The Bray-Curtis distance metric was used. The point labels include the subject number followed by the type of sample. NS microcosms (*NS*) are shown in *green*, and WS microcosms (*WS*) are shown in *red*. The green and *red ellipses* are arbitrary and provided solely to aid visualization of each group

Additional file 9 provides the complete list of spectral count assignments to SEED proteins. The SEED analysis was able to assign functional roles to 50 % of 1,126,203 total spectra, representing 1969 distinct proteins across all 12 NS and WS pairs. The SEED PCoA plot (Fig. 4) suggested that the NS and WS microcosms were more tightly clustered on the basis of function than the case with the taxonomy PCoA plot in Fig. 3. Almost complete separation was seen along coordinate 1, with the exceptions of 730 NS and 733 NS. The NS microcosms continued to show dispersion along coordinate 2, whereas most WS microcosms were tightly clustered towards the center of that axis (733 NS was an outlier with respect to coordinate 2).

Hierarchical clustering results were fully consistent with the spatial distribution of samples in the PCoA plot. In contrast to the results for the HOMINGS and LCA taxonomy analyses, extensive differences in the protein relative abundance profiles for the two major clusters were apparent in the SEED heat map (Additional file 10).

edgeR analysis indicated that 505 proteins (26 % of the 1969 assigned) differed between the NS and WS microcosms at FDR ≤ 5 % (Additional file 11). By that criterion, functional analysis was much more successful than either the HOMINGS or LCA taxonomic analyses at identifying differences between the NS and WS microcosms. In other words, sucrose pulsing induced similar changes in protein relative abundance among microcosms that were taxonomically diverse. The following discussion describes major trends, using broadly distributed proteins (present in at least 12 samples), which differed greatly between NS and WS microcosm pairs (FDR ≤ 5 %), as representative examples (Table 4). The MetaCyc Metabolic Pathway Database was the primary reference for protein function in NS microcosms [41], except as noted.

The NS relative abundance pattern was consistent with mucin degradation (ABC transporter components for galactose and dipeptides), with mixed acid fermentation directed towards the generation of formate, acetyl CoA, and acetate by various pathways (pyruvate formate-lyase, acetate kinase, acetaldehyde dehydrogenase). Amino acid degradation leading to the release of ammonia was also a prominent feature (NAD-specific glutamate dehydrogenase, lysine 2,3-aminomutase, tryptophanase, histidine ammonia-lyase). All of those proteins were significantly upregulated in NS microcosms at FDR ≤ 5 %. Those mechanisms are likely to have contributed to the maintenance of NS microcosms at a stable pH between 6.0 and 7.0. The arginine deiminase pathway has been suggested to participate in ammonification of oral biofilms [13, 42]. Arginine deiminase and carbamate kinase were elevated in NS at FDR ≤ 5 %. Ornithine carbamoyltransferase showed a similar trend (FDR = 0.19).

Our LCA results indicated that *S. mutans* was undetectable in 8 WS microcosms and a minor component of the remaining 4 WS microcosms (see above). Nevertheless, most studies of oral microbial responses to sucrose and pH stress have focused on *S. mutans*, due to its status as a presumptive caries pathogen. Accordingly, we used that literature as a knowledge base for interpretation of the WS results (none of the proteins discussed could be unequivocally assigned to *S. mutan*s at the species level).

Sucrose pulsing induced a very distinct relative abundance pattern in WS microcosms sampled at minimum pH on day 3. Multiple sucrose degradation and glycolysis enzymes were elevated at FDR ≤ 5 %, notably sucrose-6-phosphate hydrolase, fructokinase, 6-phosphofructokinase, aldolase, and enolase. Aldolase generates fructose-1,6-diphosphate, which activates l-lactate dehydrogenase [42], and both were strongly upregulated (Table 5). This suggested a shift to lactic acid as a fermentation product, which was consistent with the cyclic drops in pH that accompanied sucrose pulsing. Some proteins involved with acid tolerance were upregulated, notably GroES, DnaJ, and components of the F0F1 ATPase. Interestingly, DnaK and GroEL, which are strongly associated with *S. mutans* acid tolerance [42, 43], failed to meet the FDR ≤ 5 % criterion. This may have reflected the low relative abundance of *S. mutans* in the WS microcosms. In that context, it is worth noting that pyruvate oxidase was strongly upregulated in every WS microcosm. That enzyme is made by most oral streptococci but is absent from *S. mutans* [44, 45]. Pyruvate oxidase generates H_2_O_2_, an inhibitor of *S. mutans* growth in competition experiments [44, 45]. The iron-sulfur cluster assembly protein SufB, which is associated with protection against oxidative stress [46], likewise was strongly upregulated. This would be consistent with the presence of H_2_O_2_. All WS microcosms did drop below pH 5 at minimum pH, which suggested that non-mutans streptococci played a major role in lactate production.

**Table 5:** edgeR results for proteins discussed in the text with FDR ≤ 5 % (*): WS vs. NS microcosms

Protein assigned by MEGAN5 using SEED	logFC	adjPValue	No. present^a^
Pyruvate formate-lyase (EC 2.3.1.54)	−1.12	5.89E-06*	24
Acetate kinase (EC 2.7.2.1)	−1.41	8.71E-13*	24
NAD-specific glutamate dehydrogenase (EC 1.4.1.2)	−4.20	2.87E-13*	23
Lysine 2,3-aminomutase (EC 5.4.3.2)	−8.34	1.53E-10*	13
Tryptophanase (EC 4.1.99.1)	−6.46	1.07E-15*	19
Histidine ammonia-lyase (EC 4.3.1.3)	−1.86	2.62E-03*	22
Arginine deiminase (EC 3.5.3.6)	−0.55	4.04E-02*	24
Carbamate kinase (EC 2.7.2.2)	−0.86	6.86E-03*	24
Fructose-bisphosphate aldolase class II (EC 4.1.2.13)	1.08	8.07E-07*	24
Enolase (EC 4.2.1.11)	0.47	4.56E-02*	24
l-lactate dehydrogenase (EC 1.1.1.27)	1.90	2.98E-14*	24
Heat shock protein 60 family co-chaperone GroES	1.03	1.41E-05*	24
Chaperone protein DnaJ	1.34	1.59E-07*	24
Pyruvate oxidase (EC 1.2.3.3)	3.83	8.01E-26*	22
Iron-sulfur cluster assembly protein SufB	2.27	1.36E-14*	24
Acetolactate synthase large subunit (EC 2.2.1.6)	2.62	1.44E-05*	20
Ketol-acid reductoisomerase (EC 1.1.1.86)	1.92	2.54E-17*	24
Branched-chain amino acid aminotransferase (EC 2.6.1.42)	0.96	2.65E-04*	24
NADP-specific glutamate dehydrogenase (EC 1.4.1.4)	1.69	1.92E-05*	24
Glutamine synthetase type I (EC 6.3.1.2)	1.33	4.13E-10*	24

^a^Number of microcosms in which reads were assigned to that protein

Branched-chain amino acid synthesis has been identified as an acid tolerance mechanism in *S. mutans* [47, 48]. Components of that system were significantly upregulated in all WS microcosms (acetolactate synthase, ketol-acid reductoisomerase, branched-chain amino acid aminotransferase), which suggests that it operates broadly across non-mutans streptococci. Another *S. mutans* acid response mechanism involves glutamate synthesis [49, 50]. The NADP-specific glutamate dehydrogenase was very strongly upregulated in all WS microcosms. By contrast, the NAD-specific glutamate dehydrogenase predominated in all NS microcosms. Co-factor specificity affects the direction of the reaction. The NAD-specific form degrades glutamate and generates ammonia, whereas the NADP-specific form uses ammonia to synthesize glutamate [51]. Thus, that form has the combined potential to lower the pH via assimilating ammonia, while simultaneously upregulating other acid tolerance systems. Glutamine synthetase likewise was strongly upregulated in all WS microcosms, which further promotes ammonia assimilation. Both enzymes are part of the GlnR regulon, which appears to play a strong role in *S. mutans* acid tolerance [50]. The WS microcosm results suggest that this mechanism may also be broadly conserved in other streptococci.

## Conclusions

Our oral microcosm biofilm model of sucrose-induced dysbiosis has provided multi-omic data that support the “conservation of function” concept. The real-time pH curves confirmed that the NS and WS communities demonstrated collective pH phenotypes that were very different between groups, but very similar within groups. Two independent approaches to taxonomic analysis (HOMINGS and MEGAN5 LCA) showed that microcosm communities that retained extensive individual variation in community structure could generate similar NS and WS pH phenotypes.

By contrast, the SEED analysis showed characteristic NS and WS patterns of protein relative abundance that were highly conserved across taxonomically diverse communities. Moreover, many of the proteins that differed between each pH phenotype had functions that would act to promote maintenance of neutral pH under NS conditions or acid production and tolerance under WS conditions.

Since each NS and WS microcosm pair was grown from a single plaque inoculum, it appeared that sucrose pulsing was the main force driving the microcosms towards dysbiosis. All 12 inoculums were obtained from caries-active children, so we cannot rule out the possibility that the microbiotas from each subject were already predisposed to respond strongly to sucrose. Planned microcosm studies of plaque from caries-free children will help to address that question.

An obvious limitation of our microcosm model is that it cannot replicate all aspects of the oral environment. However, the deep level of metaproteomic analysis it allows makes it suitable as a platform for discovering which proteins are most consistently abundant during dysbiosis. Targeted proteomic approaches then can be used to determine whether those proteins are also abundant when plaque is exposed to sucrose in the mouth. In that case, it may be possible to define a set of dysbiosis biomarkers that could be used to detect at-risk tooth surfaces before the development of overt carious lesions.

## Methods

### Collection and processing of saliva and plaque samples

Our sample collection and processing protocol was the same as described in a previous publication [23]. Briefly, samples were collected by a pediatric dentist from 12 children with mixed dentition (ages 6–11.5 years). Previous restorations were present in all children, and all were deemed by the clinical examiner to be at high risk for future caries. Active carious lesions were present in all subjects except 730 and 852 (Additional file 12). None of the subjects had taken antibiotics within 3 months prior to sample collection.

Children were asked to expectorate resting whole saliva into ice-cooled tubes. The dentist collected plaque from the margins of a single existing composite restoration in a primary tooth from each child (see clinical data in Additional file 12). A sterile instrument was used, and samples were immediately deposited into a vial containing pre-reduced anaerobic transfer medium. The University of Minnesota Institutional Review Board approved all procedures involving human subjects.

Each saliva sample was clarified by centrifugation, diluted twofold in a buffer simulating the ionic composition of saliva, and then filter-sterilized. Each matching plaque sample was dispersed by vortexing, and a portion was retained for DNA extraction and HOMINGS analysis (as described below).

### Oral microcosm biofilm model

The remainder of each plaque suspension was incubated in paired CDC biofilm reactors, according to our published protocol [23]. Briefly, hydroxyapatite disks were placed into sample holders for each reactor. Pellicles were formed, by coating each disk with processed saliva from a single child. Each set of coated disks then were inoculated with the plaque suspension from the corresponding child, placed into reactors containing 350 ml basal mucin medium (BMM) [25], and incubated at 37 °C under constant shear (125 rpm) for 24 h. BMM was then flowed through one reactor at 17 ml/min (125 rpm; 37 °C) for 48 h (NS conditions).

The second reactor additionally was sucrose-pulsed five times per day (20 *v*/*v*%, 43 ml each time) analogous to three meals and two snacks for the second and third day (the flow rate for the second reactor was set at 20 ml/min, to reduce fouling). Sucrose pulsing was discontinued at night.

Real-time pH was recorded every 15 min, throughout the 72-h incubation (NS and WS conditions). On the third day around 4:00 PM (the time when the sucrose-pulsed reactor typically reached minimum pH), biofilms from multiple disks per reactor then were pooled to create NS and WS microcosm samples. This process was repeated until paired NS and WS samples had been obtained for each of the 12 children. A portion of those samples was processed for DNA extraction, and the remainder was used for protein extraction.

### DNA extraction and HOMINGS analysis

DNA was extracted using the protocol described on the HOMINGS website. The extracts then were shipped on dry ice to the HOMINGS core facility at the Forsyth Institute. Samples were processed through the HOMINGS workflow, which involves PCR amplification using universal primers for the V3–V4 regions of 16S rRNA, barcoded multiplex Illumina MiSeq sequencing, demultiplexing, and bioinformatic analysis with QIIME and ProbeSeq, a program developed at Forsyth to screen fastq files for sequences that match a validated set of probes for 638 species-level targets representing 538 oral species present in the HOMD database (plus an additional 129 genus-level probes). Additional information about HOMINGS, including validation, calibration, and reproducibility data, is available on the HOMINGS website [52]. Submission of a methods article with a more detailed description of the HOMINGS approach is planned for November 2015 (personal communication, Dr. Bruce Paster, The Forsyth Institute).

### Protein extraction from microcosm biofilms

An unanticipated challenge in developing the protein extraction protocol was that it was much more difficult to extract proteins from sucrose-pulsed biofilms. Initial yields from WS samples were much lower, and extracts also contained contaminants that interfered with mass spectrometry. It seemed likely that biofilm matrix components were not being adequately removed. A protocol combining highly denaturing conditions and pressure-cycling technology was developed to address the refractory nature of protein extraction from WS biofilms. Pressure cycling is proven to increase yields for downstream proteomics analysis [53]. It greatly improved protein recovery from both NS and WS samples, so we used it consistently for both types of sample. Biofilms were snap-frozen and stored at −80 °C until needed. We ground the frozen biofilm with a mortar and pestle on dry ice and weighed the samples in 1.5-ml microfuge tubes. We added protein extraction buffer (7 M urea, 2 M thiourea, 0.4 M trietihylammonium bicarbonate, 20 % methanol, and 4 mM TCEP) in a ratio of 10 μl extraction buffer per milligram of biofilm. We sonicated the samples on ice at 30 % amplitude for 7 s with a Branson digital sonifier 250 (Branson Ultrasonics, Danbury, CT). We then pressure cycled the samples in a Barocycler NEP 2320 (Pressure Biosciences Inc., South Easton, MA) at 37 °C for 40 cycles of 35 kpsi for 30 s, followed by 0 kpsi for 15 s. We transferred the samples to new 1.5-ml microfuge tubes and added methyl methanethiosulfonate at an 8-mM final concentration to alkylate cysteines, and we incubated the samples for 15 min at room temperature. We centrifuged the samples at 10,000×*g* to spin out any insoluble material and pipetted two aliquots of each sample for Bradford assay. Once the concentration of each sample was determined, we aliquoted 150 μg of each sample for in-solution digestion. For the in-solution proteolytic digestion, we diluted the samples fourfold with ultra pure water and trypsin was added in a 1:35 ratio of trypsin—total protein. We incubated the samples at 37 °C for 16 h, and then we froze the samples and dried them *in vacuo*. We performed solid phase extraction on the samples with 3 cm^3^ Oasis MCX cartridges (Waters Corporation). The eluted peptides were dried down *in vacuo*.

### 2D liquid chromatography-mass spectrometry analysis

We processed the complex peptide mixtures by 2D LC-MS/MS. The first dimension offline HPLC system was a Shimadzu Prominence HPLC system (Shimadzu, Columbia, MD), and the HPLC column was a Phenomenex Kinetex® 5 μm EVO C18 100 Å, LC Column 150 × 2.1 mm with a Phenomenex SecurityGuard™ Gemini-NX C18 cartridge. Prior to loading, we resuspended each sample in 100 mM ammonium formate pH 10, 98 % water and 2 % acetonitrile. Buffer A was 20 mM ammonium formate, pH 10 in 98:2 water to acetonitrile, and buffer B was 20 mM ammonium formate, pH 10 in 10:90 water to acetonitrile. The flow rate was 200 μl/min with a gradient from 0–30 % buffer B over 55 min, followed by 30–60 % over 15 min. Fractions were collected every 2 min and UV absorbances were monitored at 215 and 280 nm. Peptide containing fractions was divided into two equal numbered groups, “early” and “late”. The first early fraction was concatenated with the first late fraction until all fractions were mixed together [54]. We dried the concatenated samples in vacuo. After the first dimension peptide separation, we processed the dried peptide pellets according to the Stage Tip protocol [55], with the following revisions: the 3M (St Paul, MN) Empore™ solid phase extraction disks were styrenedivinylbenzene-reversed phase sulfonate (SDB-RPS), the peptides were reconstituted in aqueous 0.2 % formic acid, membranes were conditioned with acetonitrile and then ultrapure water, and wash solvent 1 was 95:5:0.2 %, water to acetonitrile to formic acid (FA). Wash solvent 2 was acetonitrile, and elution solvent was 60:35:5 %, acetonitrile to water to ammonium hydroxide. We dried the eluted peptides in a speed vacuum. We dissolved the dried peptide pellets in 98:2:0.1 %, water to acetonitrile to trifluoroacetic acid and analyzed by capillary LC-MS/MS on a Velos Orbitrap system according to the previously published LC and MS methods [56], with the following exceptions: lock mass was not enabled, dynamic exclusion setting list size was 200 values, duration was 30 s, and mass width was +/− 10 ppm.

### Metaproteomic workflow

**Fig. 5:**
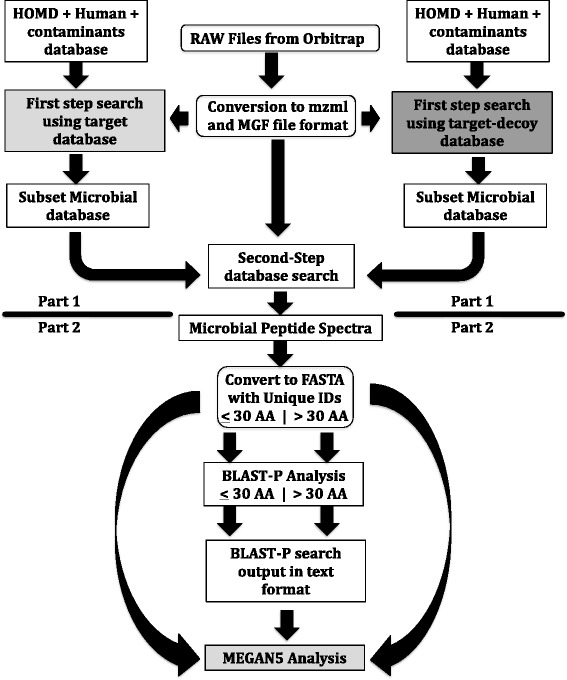
Simplified Galaxy-P workflow. In Part 1, RAW files for each NS and WS pair were run in parallel within a single workflow to generate a second-step search database for each pair. In Part 2, the spectra for the NS and WS samples then were searched independently and the search outputs were processed to generate inputs for MEGAN analysis

A customized automated workflow was developed within the Galaxy-P platform (Fig. 4). Multiple Xcalibur .RAW data files for each 2D LC-MS analysis were converted into mzml files (using msconvert tool within Galaxy-P) and then converted into .MGF files (using MGF formatter tool within Galaxy-P) for ProteinPilot analysis [57]. The files were searched using our published two-step strategy [30–33], which has also been used in metaproteomic studies by independent laboratories [58]. The first search was carried out against a merged target dataset, which combined the translated version of the Human Oral Microbiome Database (HOMD) genomic dataset (downloaded on April 29, 2013) with the UniProt human protein dataset (background database) with the protein contaminants database (common repository adventitious proteins from the GPM database). This was followed by a target-decoy search of the merged dataset. The peptide summary files from both searches were parsed out for peptides with accession numbers associated with the HOMD dataset. Those entries were combined and merged once more with the Human UniProt and contaminant peptide datasets. This “second-step dataset” was searched using the target-decoy database search strategy with ProteinPilot, and peptide-spectrum matches (PSMs) with a confidence score at a 5 % target-decoy search local false discovery rate (FDR) were used for further analysis . All instances of the spectrum for a given peptide were retained, to allow for spectral counting. Text-formatting tools within Galaxy-P were used to generate a FASTA list with a unique identifier for every peptide spectrum (required by MEGAN5) (Fig. 5). That file was split into a list of short sequences (≤30 amino acids), and a list of long sequences (>30 amino acids). This was done so that the short sequences file could be searched against the BLAST-NR database, using the default short input sequence parameters for BLAST-P (the long sequences files were searched using the regular default parameters for BLAST-P). Finally, the short and long BLAST-P output files were merged and exported from Galaxy-P (along with the full Unique ID FASTA reads file).

The BLAST-P and Unique FASTA reads files for each sample were imported into the MEGAN5.7.1 software package [59] with input parameters set to Minimum BLAST Bit Score = 30, Maximum BLAST Expected Value = 3.0, Top Percent = 10. Minimum Support Percent = 0.0 (off), Minimum Support = 5, LCA Percent = 100, Minimum Complexity = −1.0 (off), and Use Minimal Coverage Heuristic = On. The BLAST-P file was parsed to extract phylogenetic and functional information for each metaproteome. Separate MEGAN5 .rma files were generated for each NS and WS samples from each subject. The MEGAN5 compare option then was used to generate a comparison file incorporating all 24 data sets. Spectral counts for each taxon and protein detected then were exported for statistical analysis in the R statistical computing environment [60].

### Statistical analysis

Bray-Curtis distance matrices were calculated for the HOMINGS, MEGAN LCA, and MEGAN SEED datasets using functions available in the vegan package. Those were used as input for PCoA and hierarchical clustering.

The PCoA plots were generated using Kruskal’s non-metric multidimensional scaling algorithm (as implemented in the isoMDS function in the MASS package). Corresponding heat maps were generated using the heatmap.2 function from the gplots package. The linear model used with the edgeR software included within-subject effects for sucrose pulsing (NS vs. WS) and individual level. We first fitted a common dispersion parameter (using the function estimateGLMCommonDisp), then estimated how these estimates depend on the mean (using the function estimateGLMTrendedDisp), and finally smoothed the individual level dispersion parameters towards the common dispersion parameter in a data dependent fashion using an empirical Bayes approach (using the function estimateGLMTagwiseDisp). The *p* values from the tests at the individual protein/species level were then adjusted using the Benjamini-Hochberg procedure, controlling the FDR at 5 %.

## Availability of supporting data

Multiplexed FASTQ output files for the first step in the HOMINGS analysis with barcode lists and an outline of the subsequent workflow are available at https://drive.google.com/folderview?id=0B610-sFuW0BKNUp0aHg2dHJGdlU&usp=sharing (note that samples were run in two batches). The mass spectrometry proteomics data have been deposited to the ProteomeXchange Consortium (http://proteomecentral.proteomexchange.org) via the PRIDE partner repository [61] with the dataset identifier PXD003151. HOMINGS probe counts, MEGAN5 LCA species-level spectral counts, SEED protein spectral counts, edgeR output files, and subject clinical metadata supporting the results of this article are included with the article as additional files.

## Additional files

Additional file 1:
**ProbeSeq results as received from the HOMINGS core facility.** The first worksheet provides read count summaries for each sample. The second worksheet lists read counts for all HOMINGS probes (including those not detected) for each sample. The third worksheet presents the read counts as a percent of total reads for each sample, and the fourth worksheet summarizes results for each sample as stacked column plots. Additional file 2:
**Heat map of HOMINGS data.** The color key is on a logarithmic scale. The Bray-Curtis distance metric was used to generate the dendrograms. Each column represents a plaque, NS, or WS microcosm sample; labels provided for the columns are colored blue for plaque, green for NS, or red for WS. Each row represents one of the 453 HOMINGS probes that were positive for at least one sample (probes that were not detected in any sample are excluded). Row labels are not shown, since they would not be legible. Additional files 3, 4, and 5 list all positive probes. Additional file 3:
**Complete edgeR results for HOMINGS taxon probes, comparing Plaque vs. NS pairs.** Each row summarizes the results for a HOMINGS taxon probe that was detected in at least one plaque or NS sample. The rows were first sorted into two groups (using column G), those with an adjusted *p* value ≤0.05 (shown in blue) and those in which the adjusted *p* value exceeded that threshold. Within each group, the rows then were sorted according to the number of plaque samples in which reads for a probe were ≥1 % (as a percent of total reads), in descending order (using column H). The taxon probe names in the FDR ≤ 5 % group were shaded red when the log fold change value was positive (P > NS), and green when the log fold change value was negative (NS > *P*). Additional file 4:
**Complete edgeR results for HOMINGS taxon probes, comparing Plaque vs. WS pairs.** The spreadsheet otherwise is formatted in the same way as Additional file 3. Additional file 5:
**Complete edgeR results for HOMINGS taxon probes, comparing WS vs. NS pairs.** There were no probes with an adjusted *p* value ≤0.05, so the rows only were sorted according to the number of samples in which the relative abundance of a taxon probe exceeded 1 % of total reads (in descending order, using column H). Additional file 6:
**Counts for spectra assigned at the species level by the MEGAN5 LCA.** Each row lists spectral counts assigned to a NCBI species level taxon in at least one NS or WS sample. The columns are paired, so that each NS sample is matched with its WS counterpart. The rows are sorted in descending order by the number of samples in which that species was assigned (using column Z). Additional file 7:
**Heat map of species-level MEGAN5 LCA assignments.** The color key is on a logarithmic scale. The Bray-Curtis distance metric was used to generate the dendrograms. Each column represents a NS, or WS microcosm sample; labels provided for the columns are colored green for NS or red for WS. Each row represents one of the 303 LCA-assigned species. Row labels are not shown, since they would not be legible. Additional file 6 lists all species assignments. Additional file 8:
**Complete edgeR results for spectra assigned at the species level by the MEGAN5 LCA, comparing WS vs. NS pairs.** The species names assigned by MEGAN5 were derived from the NCBI taxonomy database. The spreadsheet otherwise is formatted in the same way as Additional file 3. Additional file 9:
**Counts for spectra assigned to SEED ontology proteins by MEGAN5.** Each row lists spectral counts assigned to a SEED protein. The spreadsheet otherwise is formatted in the same way as Additional file 6. Additional file 10:
**Heat map of MEGAN5 SEED assignments.** The color key is on a logarithmic scale. The Bray-Curtis distance metric was used to generate the dendrograms. Each column represents a NS, or WS microcosm sample; labels provided for the columns are colored green for NS or red for WS. Each row represents one of the 1969 SEED-assigned proteins. Row labels are not shown, since they would not be legible. Additional file 9 lists all protein assignments. Additional file 11:
**Complete edgeR results for spectra assigned to SEED proteins by MEGAN5, comparing WS vs. NS pairs.** The proteins names assigned by MEGAN5 were derived from the SEED ontology database. The spreadsheet is formatted in the same way as Additional file 8, except that column H sorts the proteins by the number of microcosms in which they were detected (in descending order). Additional file 12:
**Clinical metadata for subjects.** Lists each subject’s age at time of sampling, gender, the universal ID code for the restored primary tooth from which the sample was taken and the clinical characteristics of the composite restoration around the margins of which plaque was collected.
