# Supplementary figures and images for: Protein relative abundance patterns associated with sucrose-induced dysbiosis are conserved across taxonomically diverse oral microcosm biofilm models of dental caries
Source: Microbiome. 2015 Dec 19;3:69. doi: 10.1186/s40168-015-0136-z (PMC4684605; doi:10.1186/s40168-015-0136-z)

Color Key  
and Histogram

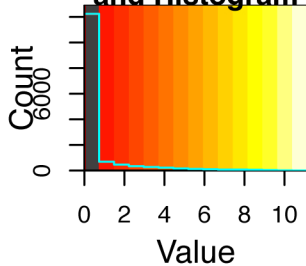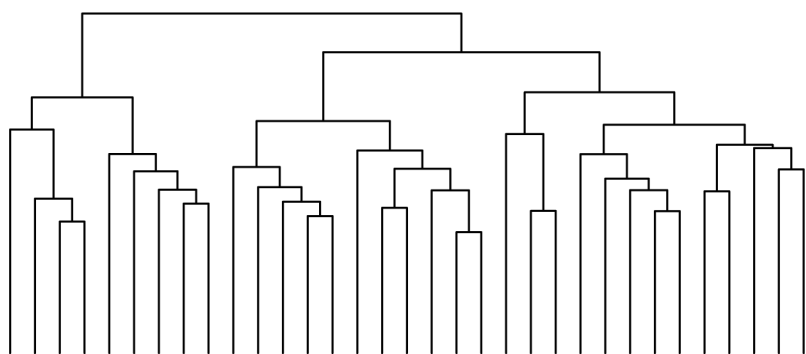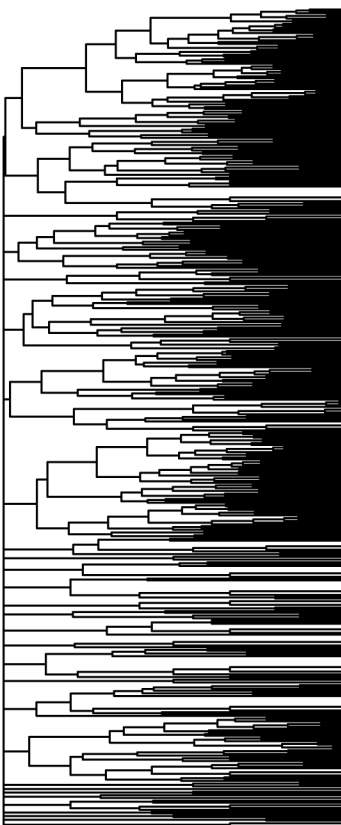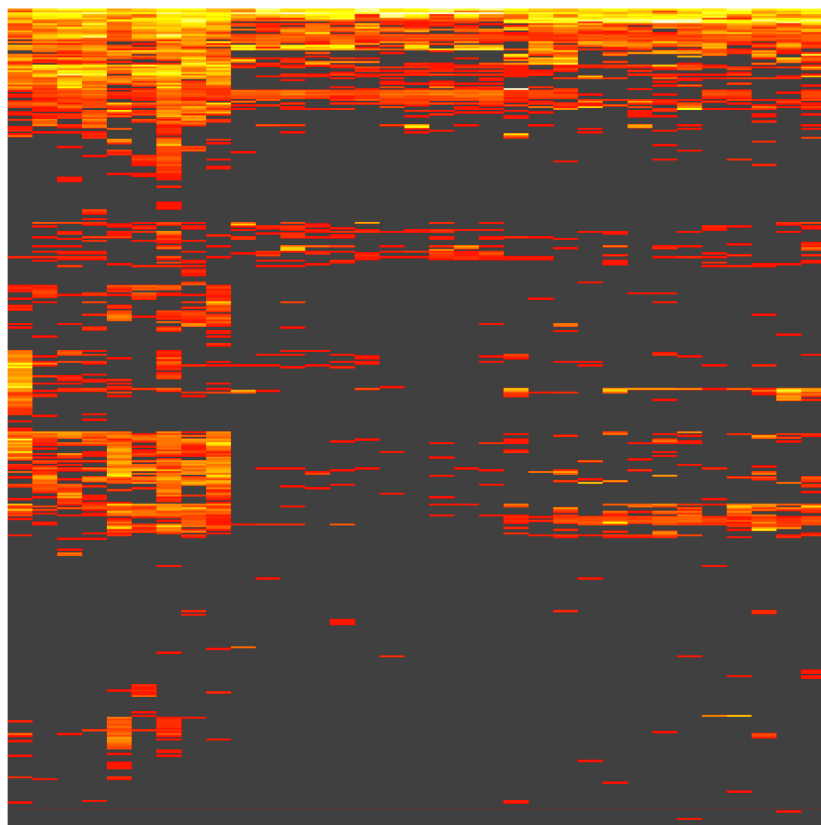

781P  
734P  
769P  
861P  
737P  
852P  
866P  
730P  
760P  
795WS  
730WS  
734WS  
737WS  
867WS  
733NS  
769WS  
861WS  
733WS  
866WS  
781WS  
781NS  
760WS  
760NS  
730NS  
867NS  
861NS  
866NS  
769NS  
852WS  
852NS  
737NS  
795NS  
734NS

Supplement: Additional file 2: — Heat map of HOMINGS data. The color key is on a logarithmic scale. The Bray-Curtis distance metric was used to generate the dendrograms. Each column represents a plaque, NS, or WS microcosm sample; labels provided for the columns are colored blue for plaque, green for NS, or red for WS. Each row represents one of the 453 HOMINGS probes that were positive for at least one sample (probes that were not detected in any sample are excluded). Row labels are not shown, since they would not be legible. Additional files 3, 4, and 5 list all positive probes. [file 40168_2015_136_MOESM2_ESM.pdf]

Color Key

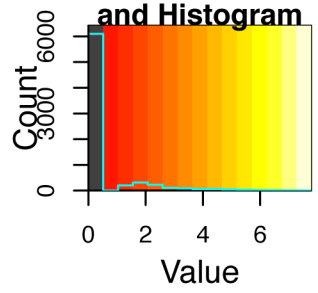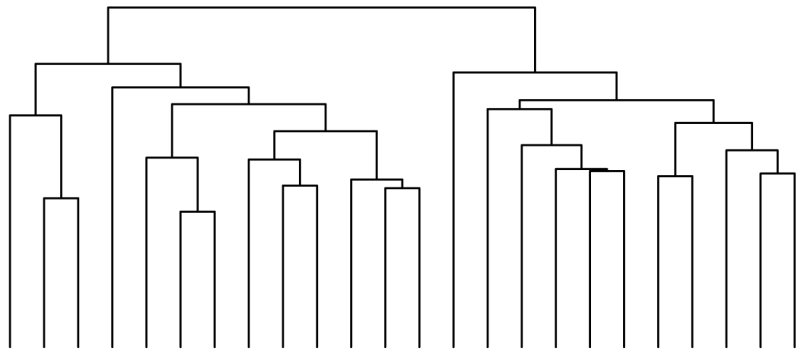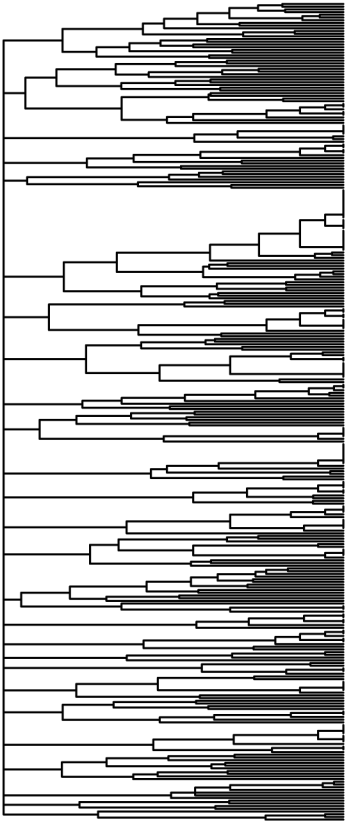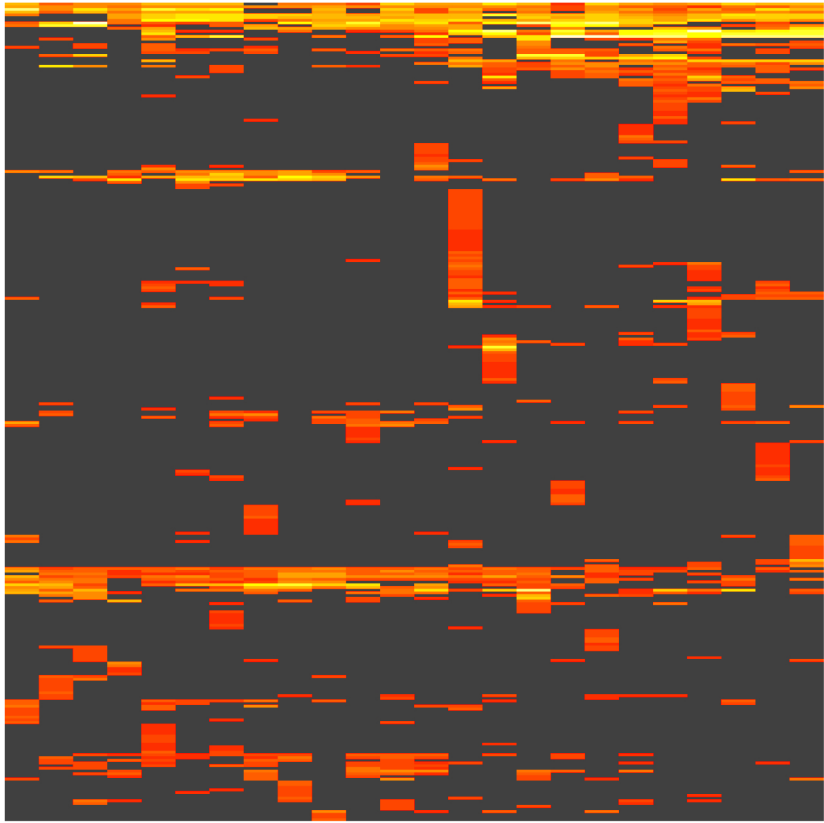

730WS  
733WS  
733NS  
734WS  
852WS  
737WS  
867WS  
795WS  
781WS  
861WS  
866WS  
769WS  
760WS  
852NS  
781NS  
730NS  
866NS  
861NS  
769NS  
760NS  
737NS  
795NS  
867NS  
734NS

Supplement: Additional file 7: — Heat map of species-level MEGAN5 LCA assignments. The color key is on a logarithmic scale. The Bray-Curtis distance metric was used to generate the dendrograms. Each column represents a NS, or WS microcosm sample; labels provided for the columns are colored green for NS or red for WS. Each row represents one of the 303 LCA-assigned species. Row labels are not shown, since they would not be legible. Additional file 6 lists all species assignments. [file 40168_2015_136_MOESM7_ESM.pdf]

Color Key

and Histogram

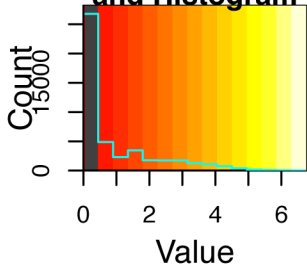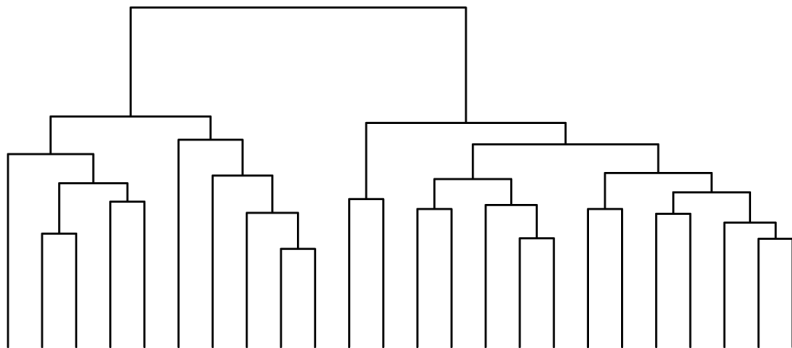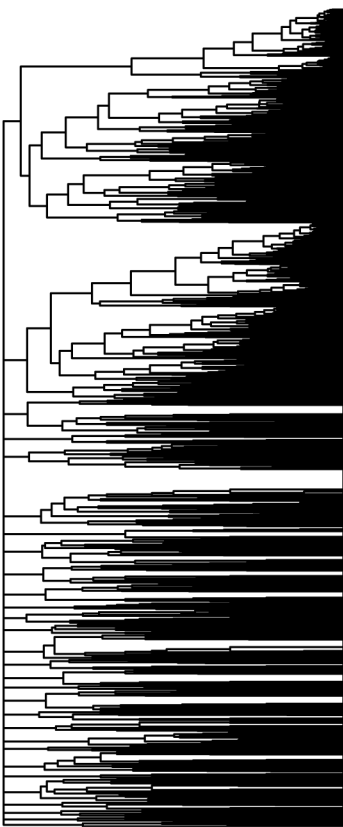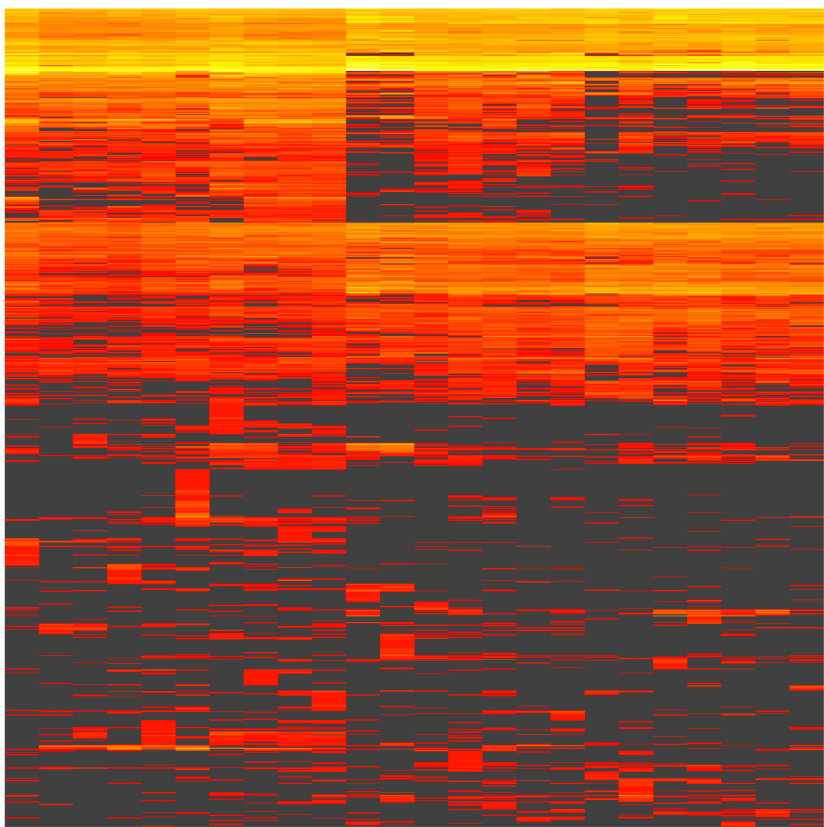

781NS  
795NS  
734NS  
737NS  
867NS  
852NS  
861NS  
866NS  
760NS  
769NS  
733WS  
733NS  
730NS  
760WS  
852WS  
867WS  
737WS  
730WS  
769WS  
781WS  
866WS  
861WS  
795WS  
734WS

Supplement: Additional file 10: — Heat map of MEGAN5 SEED assignments. The color key is on a logarithmic scale. The Bray-Curtis distance metric was used to generate the dendrograms. Each column represents a NS, or WS microcosm sample; labels provided for the columns are colored green for NS or red for WS. Each row represents one of the 1969 SEED-assigned proteins. Row labels are not shown, since they would not be legible. Additional file 9 lists all protein assignments. [file 40168_2015_136_MOESM10_ESM.pdf]
